# Perforator Relocation in Free Style Local Perforator Flaps

**Published:** 2013-01

**Authors:** Nikhil Panse, Parag Sahasrabudhe, Namrata Joshi

**Affiliations:** 1 Department of Plastic Surgery, BJ Medical College and Sassoon Hospital, Pune, India;; 2 Department of Surgery, BJ Medical College and Sassoon Hospital, Pune, India

**Keywords:** Perforator relocation, Free style local perforator, Flap

## Abstract

Local perforator flaps have evolved over the time to provide complete and stable coverage of soft tissue defects. Technical advances and experience with the perforator flaps have allowed the harvest of local perforator flaps in a free style manner by offering greater freedom in flap selection. We have proposed a technical modification in the harvest of a perforator flap by relocating the perforator to an anatomically favorable location. This has led to decreased traction over the perforator and provided some amount of added length to the perforator.

## INTRODUCTION

The concept of free-style local perforator flap surgery represents a safe, versatile and reliable treatment option in the management of soft-tissue defects.^1^ Free style local perforator flaps are now accepted as one of the established treatment options for coverage of soft tissue defects.^2^ This case is presented to highlight a technical modification to relieve stretch on the perforator, and increase the reach of the local free style perforator flap.

## CASE REPORT

**Fig. 1:**
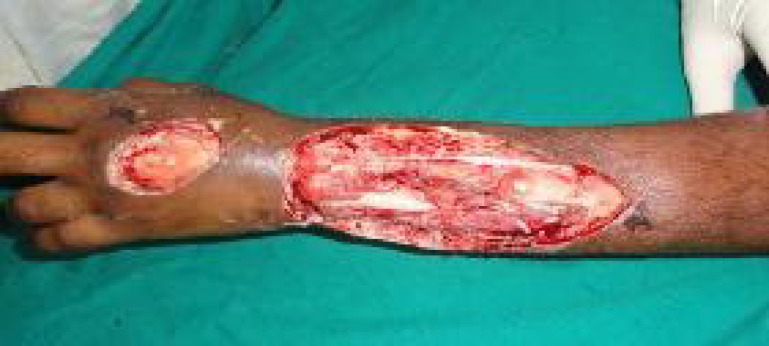
Actual size of the defect after debridement

Patient presented with post snakebite defect on the dorsum of forearm. A thorough debridement of all macroscopically infected and non-viable tissues resulted in exposed ulna on the distal dorsoulnar border of the forearm. An ulnar artery perforator flap was planned for the defect (Figure 1).

**Fig. 2:**
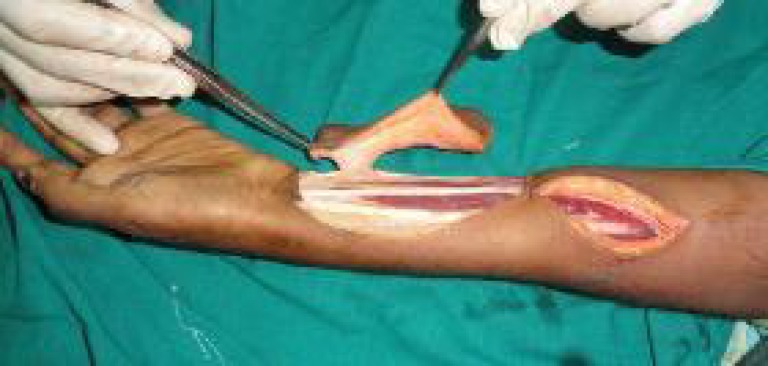
Flap elevation showing the elegant pedicle in the radial side of FUC

An exploratory incision was made, and a perforator of the ulnar artery was identified in the vicinity of the defect, away from the zone of trauma. After the perforator was identified, it was noted that the perforator was on the radial side of the Flexor carpi ulnaris (FCU), and the defect was on the ulnar side of the FCU (Figure 2).

The flap needed a transposition type of movement, and not rotation around the axis like a propeller flap. A small fascial strand was kept along with for support. Intraoperative handling of the perforator and prevention of drying and spasm of the perforator was done by constant irrigation with lignocaine solution as done by us routinely while operating perforator flaps.^1^^,^^2^

**Fig. 3:**
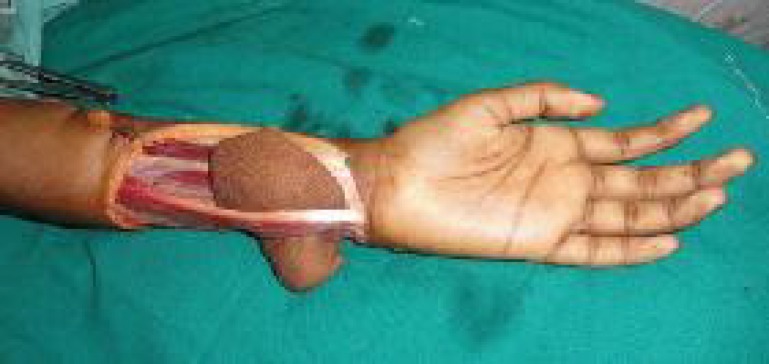
Transfer of the flap to proper position

Although the flap was comfortably covering the defect, the perforator had to course over the FCU. This lead to a minimal traction on the perforator. To minimize the traction, we relocated the perforator to an anatomically favorable location- the ulnar side of the FCU (Figure 3).

An incision was made over the paratenon of the FCU on its poster lateral aspect in a manner which would prevent the tendon exposure at the time of grafting. The musculotendinous junction was selectively lifted sufficiently enough so that the flap could be tunneled beneath the flexor carpi ulnaris.

**Fig. 4:**
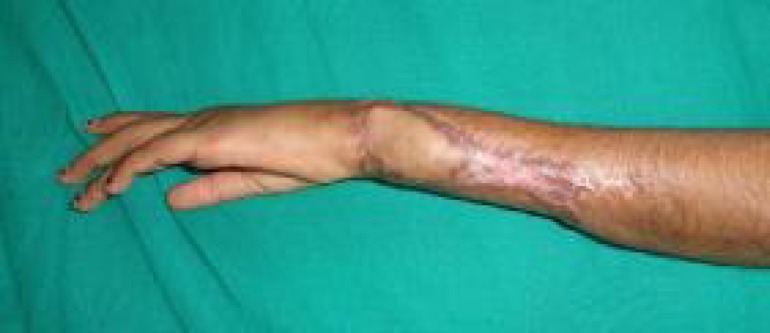
End result with acceptable coverage and view

After adequate tunnel was created, the flap along with the perforator was relocated from the radial side of the FCU to the ulnar side of the FCU. This minimized the traction over the perforator. And the flap was able to achieve an added distance of around 0.5 to one centimeters. The flap was comfortably inset over the defect, and donor area grafted (Figures 1-4) 

## DISCUSSION

The local perforator flaps offer a movement depending on position of the perforator, tissue elasticity and perforator vessel length. When performing a local free style perforator flap, it is important to obtain sufficient vessel length of the perforator. Experimental studies revealed that longer pedicles are less sensitive to twisting forces since the length of a vessel^l^ is inversely proportion to the critical angle of twisting (DT): The latter can be increased through perforator dissection into the fascia and/or the muscle.^3^^-^^6^ In a perforator flap, regardless of the type of flap movement carried out, any stretching of the perforator vessels should be avoided to minimise the risk of vascular complication (e.g., blood-flow turbulences, endothelium alteration and platelet aggregation).^7^

We propose another method of giving some additional length to the perforator and minimize traction over the perforator. We would like to call it as relocation of the perforator. To the best of our knowledge and literature search, this has not yet been reported in literature. We feel that concept of relocating the perforator in free style local perforator flaps is especially useful in areas near the joints, where there are tendon insertions, and perforators from adjoining vessels may course on either side of the tendons. Relocation of the perforator from one side of a tendon to the other side is a safe and effective method to give a small but definite increase in length to the perforator. 

It also aids in preventing traction to the perforator when the perforator has to course over the particular tendon. Gentle handling of the perforator, prevention of traction, compression and spasm of perforator while tunneling it beneath a particular tendon are maneuvers which are critical for the favorable outcome of the flap. In our view, this relatively easy technical addition to the harvestation of free style perforator flaps will be of help to reduce traction on the perforator and provide some additional length in select cases.

## CONFLICT OF INTEREST

The authors declare no conflict of interest.

## Footenote

Please cite this paper as:

Panse N, Sahasrabudhe P, Joshi N. Perforator Relocation in Free Style Local Perforator Flaps. World J Plast Surg 2013;2(1): 47-49.
